# Clinical Outcomes of Chronic Airway Infection in Primary Ciliary Dyskinesia

**DOI:** 10.1002/ppul.71828

**Published:** 2026-09-06

**Authors:** Thomas G. Saba, Madsen Zimbric, Harlan McCaffery, Lindsay J. Caverly

**Affiliations:** ^1^ Department of Pediatrics University of Michigan Medical School Ann Arbor Michigan USA

**Keywords:** bronchiectasis, chronic infection, *Pseudomonas aeruginosa*

## Abstract

**Introduction:**

Primary ciliary dyskinesia (PCD) is a rare disorder of impaired respiratory mucociliary clearance and chronic pulmonary infections. The clinical impact of chronic infection is poorly understood. The objective of this single‐center, retrospective cohort study was to assess clinical outcomes of people with PCD and chronic airway infection. We hypothesized that chronic airway infection would be associated with greater declines in lung function and higher pulmonary exacerbation rates.

**Methods:**

Linear mixed effects models were used to analyze the association of chronic infection with percent predicted forced expiratory volume in 1 s (ppFEV1). Poisson mixed models were used to analyze the association of chronic infection with exacerbation rates.

**Results:**

Thirty‐two people with PCD were included: 19 (59%) with chronic infection including 5 (16%) with chronic *Pseudomonas aeruginosa* (PA), 10 (31%) with chronic *Haemophilus influenzae* (HI), 2 (6%) with chronic methicillin‐sensitive *Staphylococcus aureus* (MSSA) and 2 (6%) with chronic *Aspergillus fumigatus* (AF). A lower ppFEV1 was associated with chronic AF, but not with other chronic infections. The exacerbation rate ratio was 1.29 [95% CI 0.72, 2.30] (*p* = 0.388), 5.03 [95% CI 1.41, 18.0] (*p* = 0.013), 0.41 [95% CI 0.16, 1.08] (*p* = 0.072) and 2.19 [95% CI 0.39, 12.30] (*p* = 0.373) after development of chronic HI infection, chronic PA infection, chronic MSSA infection and chronic AF infection, respectively.

**Conclusion:**

In this single‐center, retrospective study of chronic airway infections in PCD, chronic infection with PA was associated with a greater rate of pulmonary exacerbations but ppFEV1 decline was only associated with chronic AF infection.

## 1 Introduction

Primary ciliary dyskinesia (PCD) is a congenital disorder of impaired motile cilia with an estimated incidence of 1 in 7500 [1]. In PCD, abnormal ciliary motility impairs mucociliary clearance in multiple organ systems. Approximately 80% of people with PCD have respiratory distress at birth [2], 50% have laterality defects (including 6%–12% with heterotaxy [3, 4]), 80% of men and 60% of women have subfertility [5], and nearly 40% of children experience hearing loss [6]. Chronic oto‐sino‐pulmonary infections, bronchiectasis, and chronic productive cough are universal in people with PCD (pwPCD) and are significant contributors to decreased quality of life [2, 3, 4, 5, 6]. The bacterial species most often found in PCD airway cultures are *Haemophilus influenzae* (HI), methicillin‐sensitive *Staphylococcus aureus* (MSSA), methicillin‐resistant *Staphylococcus aureus* (MRSA), *Streptococcus pneumoniae*, and *Pseudomonas aeruginosa* (PA) [2, 7].

Infection with PA affects up to 20% of children and 45% of adults with PCD [2, 8, 9, 10, 11]. Cross sectional studies have identified an association between PA infection and lower lung function [12] in pwPCD. The long‐term clinical impact of chronic infections in pwPCD, however, remains unclear. Chronic PA infection in pwPCD has been associated with worse bronchiectasis but the long‐term effect on lung function is variable [9, 13]. Only one small study has investigated PCD exacerbations in patients with chronic PA infections and found an association between chronic PA infection and increased exacerbations [9]. The paucity of studies of PA and other PCD airway infections, and inconsistent definitions of chronic infection in prior studies, have resulted in continued knowledge gaps regarding the clinical impact of chronic airway infection in PCD. Similarly, these knowledge gaps limit clinical decision making and optimal PCD infection management. Treatment of PA in PCD, although likely to be beneficial [14], varies widely across clinical centers [15].

The objective of this single‐center, retrospective study was to investigate the clinical outcomes of chronic airway infection in pwPCD. We hypothesized that chronic airway infection in pwPCD is associated with accelerated lung function decline and a greater rate of pulmonary exacerbations.

## 2 Methods

### 2.1 Study Subjects

This was a retrospective cohort study of pwPCD seen between 2016 and 2024 at the pediatric and adult clinics of the University of Michigan PCD Clinical and Research Center, established in 2016. Inclusion criteria were a diagnosis of confirmed PCD, and at least one respiratory culture result in the medical records with at least 1 year of data following the culture date. Briefly, the diagnosis of PCD was assigned if an individual met at least two of four clinical features of PCD: (1) unexplained neonatal respiratory distress; (2) year‐round daily productive cough; (3) year‐round nasal congestion; (4) and organ laterality defect, and: (1) biallelic pathogenic or likely pathogenic variants in PCD‐associated genes; and/or (2) a recognized class 1 ciliary ultrastructural defect under transmission electron microscopy (TEM). The study did not include patients with “PCD highly likely” [16]. Subjects without respiratory culture data and subjects with a gap in clinical care for greater than 1 year were excluded. The study was determined by the University of Michigan IRB to pose no more than minimal risk and informed consent was waived.

### 2.2 Microbiological Data

Respiratory cultures were obtained as part of routine clinical care during outpatient clinic visits or inpatient admissions. Culture data from sputum, oropharyngeal (OP) swabs or bronchoalveolar lavage (BAL) fluid were included. Chronic infection was defined as at least 1 positive culture in 2 out of 3 consecutive years with at least 14 days between cultures [17].

### 2.3 Clinical Data

The following clinical data were collected from the electronic medical records: year of birth, age, age at PCD diagnosis, ethnic and racial category, body mass index (BMI), situs classification (solitus/inversus/heterotaxy), PCD genotype, TEM result, use of chronic macrolide therapy, use of PA eradication therapy, use of chronic suppressive antimicrobial therapy, respiratory culture results, lung function (percent predicted forced expiratory volume in 1 s (ppFEV1)), and number of pulmonary exacerbations per year (defined as the use of systemic antibiotics prescribed in response to changes in respiratory symptoms, including increased cough, change in sputum volume and/or color, increased dyspnea, fatigue, hemoptysis, or a decline in ppFEV1 [18, 19]). Exacerbation rates were assessed with an exacerbation rate ratio (ERR), defined as the annual exacerbation rate after onset of chronic infection relative to annual exacerbation rate prior to onset of chronic infection.

### 2.4 Statistical Analysis

Analyses were performed using R version 4.4.1 and package *lme4*. Linear mixed effects models were used to estimate the effect of chronic infection status on ppFEV1, adjusting for the quadratic trend of declining ppFEV1 with age. For patients who had chronic infections, average ppFEV1 was estimated by time from the onset of first infection, controlling for the age of the patient. Repeated measures on patients were modeled using a random intercept and, when possible, a random slope for age. Annual exacerbation rate was similarly analyzed by time from onset of chronic infection using Poisson mixed models, adjusting for year of life. The effect of chronic infection was represented as an exacerbation rate ratio: the rate of annual exacerbations of subjects after onset of chronic infections to subjects before onset of chronic infections. A secondary analysis was adjusted for patients with inner dynein arm and microtubular disorganization defects (IDA/MTD) and chronic macrolide therapy.

## 3 Results

### 3.1 Demographics

A total of 32 patients (12 age ≥18 and 20 age <18) with confirmed PCD and sufficient culture and clinical data, including records of exacerbations and ppFEV1, were included in the study. Seventeen (53%) of the individuals were female, 21 (66%) were white, 4 (13%) were Black, 3 (9%) were Hispanic, and 4 (13%) were Asian. Diagnostic information, including genotype and ciliary ultrastructure, as well as additional clinical information is described in Table 1. Chronic infection distribution is described in Table 2. Only the most frequently cultured organisms are represented in Table 2. Among 776 cultures of all organisms from 32 patients, 57% were sputum, 26% were OP, and 17% were BAL. *Pseudomonas aeruginosa* (*n* = 26) was recovered from OP and sputum cultures at similar frequencies (54% vs 46%). *Haemophilus influenzae* (*n* = 70) was evenly distributed between OP and sputum (40% each) with 20% from BAL. Methicicllin‐sensitive *Staphylococcus aureus* (*n* = 30) was identified predominantly from OP cultures (70%) compared with sputum (30%). *Aspergillus fumigatus* (*n* = 10) was recovered primarily from sputum (90%) with one bronchoalveolar lavage isolate (10%).

**Table 1:** Diagnostic and clinical features of patients with confirmed PCD.

Age at time of study	Sex	Genotype^a^	Defect on TEM	Situs	Chronic macrolide therapy
	F	*ODAD4*	NA	SS	No
2	F	*DNAH5*	NA	SIT	No
3	M	*DNAH5*	NA	SS	No
**4**	**M**	***DNAH5***	**NA**	**SIT**	**No**
6	M	*DNAH5*	NA	SIT	No
**7**	**M**	***DNAH11***	**NA**	**HTX**	**No**
7	F	*CCNO*	NA	SS	No
8	F	*DNAH5* b	ODA and IDA	SIT	No
9	F	*DNAAF5*	ODA and IDA	SS	No
10	F	*DNAI1* ^b^	ODA and IDA	SS	Yes
10	F	*DNAAF5*	NA	SIT	No
11	F	*DNAH5*	ODA and IDA	SIT	Yes
11	M	*DNAH5*	ODA and IDA	SIT	Yes
12	F	*DNAH11*	Normal	SIT	No
12	M	*CCDC40*	IDA with MTD	HTX	No
14	M	*RPGR*	Normal	SS	No
15	F	*DNAAF3*	ODA and IDA	SS	Yes
16	M	*—*	ODA	SS	No
**17**	**M**	***CCDC40***	**IDA with MTD**	**SS**	**Yes**
17	M	*CCNO*	NA	SS	No
18	F	*DNAH11*	NA	SIT	Yes
18	M	*CCDC40*	IDA with MTD	SS	Yes
19	F	*CCDC39*	IDA with MTD	SS	Yes
19	F	*DNAI2*	NA	SS	Yes
20	M	*CCDC40*	IDA with MTD	HTX	Yes
21	F	*CCDC40*	IDA with MTD	SS	No
22	F	*CCDC39*	IDA with MTD	SS	Yes
23	F	*LRRC6*	NA	SS	Yes
24	F	*DNAH11*	NA	HTX	No
**36**	**M**	***CCDC65***	**NA**	**SS**	**No**
**38**	**M**	***CCDC114***	**NA**	**SIT**	**Yes**
40	M	*DNAI1*	NA	SS	No

*Note:* Patients with chronic *Pseudomonas aeruginosa* infection are highlighted in bold.

Abbreviations: HTX, heterotaxy; IDA, inner dynein arm; MTD, microtubular disorganization; ODA, outer dynein arm; SIT, situs inversus totalis; SS, situs solitus; TEM, transmission electron microscopy

^a^ All subjects had pathogenic or likely pathogenic variants in trans if autosomal recessive inheritance or pathogenic hemizygous variants if autosomonal dominant inheritance unless otherwise indicated.

^b^ Single pathogenic allele.

**Table 2:** Prevalence of chronic infections among patients with PCD (*n* = 32; 12 Adult, 20 Pediatric).

Organism	*n* (%)
Any chronic infection	19 (59)
*Haemophilus influenzae*	10 (31)
*Pseudomonas aeruginosa*	5 (16)
Methicillin‐sensitive S*taphylococcus aureus*	2 (6)
*Aspergillus fumigatus*	2 (6)

### 3.2 Association with Lung Function

**Figure 1:**
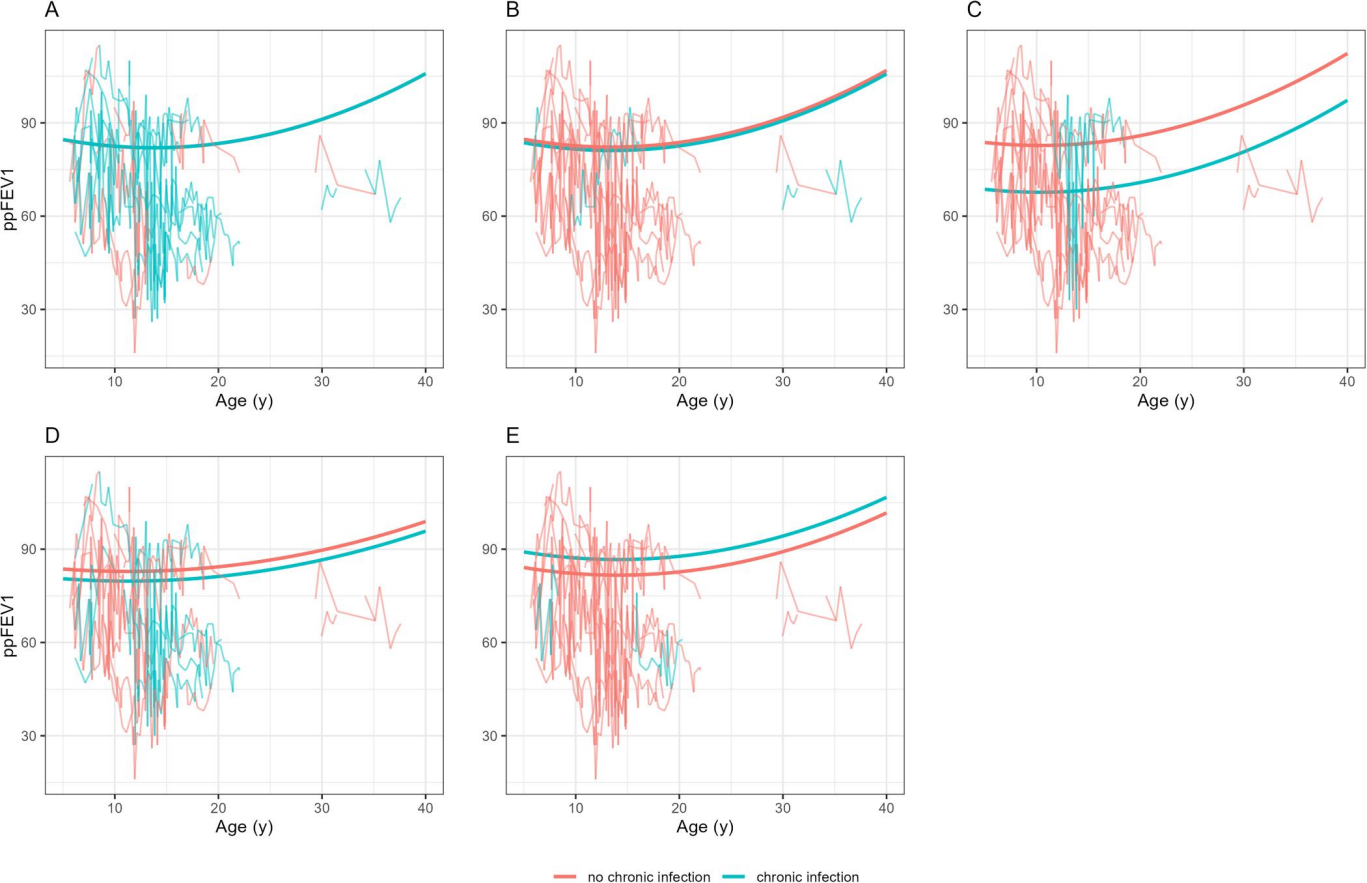
Change in ppFEV1 among patients with chronic infections. Thin lines represent observed ppFEV1 in patients without chronic infection (red) and with chronic infection (blue); thick lines represent population average estimates based on linear mixed effects models. A, any chronic infection; B, chronic *Pseudomonas aeruginosa*; C, chronic *Aspergillus fumigatus*; D, chronic *Haemophilus influenzae*; E, chronic methicillin‐sensitive *Staphylococcus aureus*. Statistically significant difference between patients with and without chronic infections was found only among those with chronic *Aspergillus fumigatus*. [Color figure can be viewed at wileyonlinelibrary.com]

Using linear mixed effects modeling, there was no statistically significant difference in ppFEV1 between pwPCD with and without chronic infection, non‐PA chronic infection, chronic HI infection, chronic PA infection, or chronic MSSA infection (Figure 1 and Table 3). There was, however, a significant difference in ppFEV1 between subjects with and without chronic AF infection (mean difference in ppFEV1 = −15.33, 95% CI [−23.54, −6.40], *p* < 0.001, *n* = 2). Very few patients had adequate ppFEV1 data recorded 3 years prior to the onset of infection, significantly limiting the ability to study lung function changes immediately before and after the onset of chronic infection.

**Table 3:** Difference in predicted lung function and exacerbation rate ratio among subjects with and without chronic infections.

Organism	Mean difference in ppFEV1 (95% CI)^a^	*p*	ERR (95% CI)*	*p*
Any chronic infection	−0.21 (−4.03, 3.66)	0.915	1.68 (1.21, 2.33)	0.002
Any non‐PA chronic infection	−0.15 (−4.18, 3.82)	0.940	1.41 (0.90, 2.20)	0.130
*Haemophilus influenzae*	−3.40 (−8.18, 1.04)	0.134	1.29 (0.72, 2.30)	0.388
*Pseudomonas aeruginosa*	−0.68 (−11.79, 11.24)	0.906	5.03 (1.41, 18.0)	0.013
MSSA	5.47 (−3.66, 14.20)	0.228	0.41 (0.16, 1.08)	0.072
*Aspergillus fumigatus*	−15.33 (−23.54, −6.40)	< 0.001	2.19 (0.39, 12.30)	0.373

ERR, exacerbation rate ratio; MSSA, Methicillin‐sensitive *Staphylococcus aureus*; PA, *Pseudomonas aeruginosa.*

^a^ Adjusted for patient age and controlling for repeated measures.

### 3.3 Exacerbation Rates

**Figure 2:**
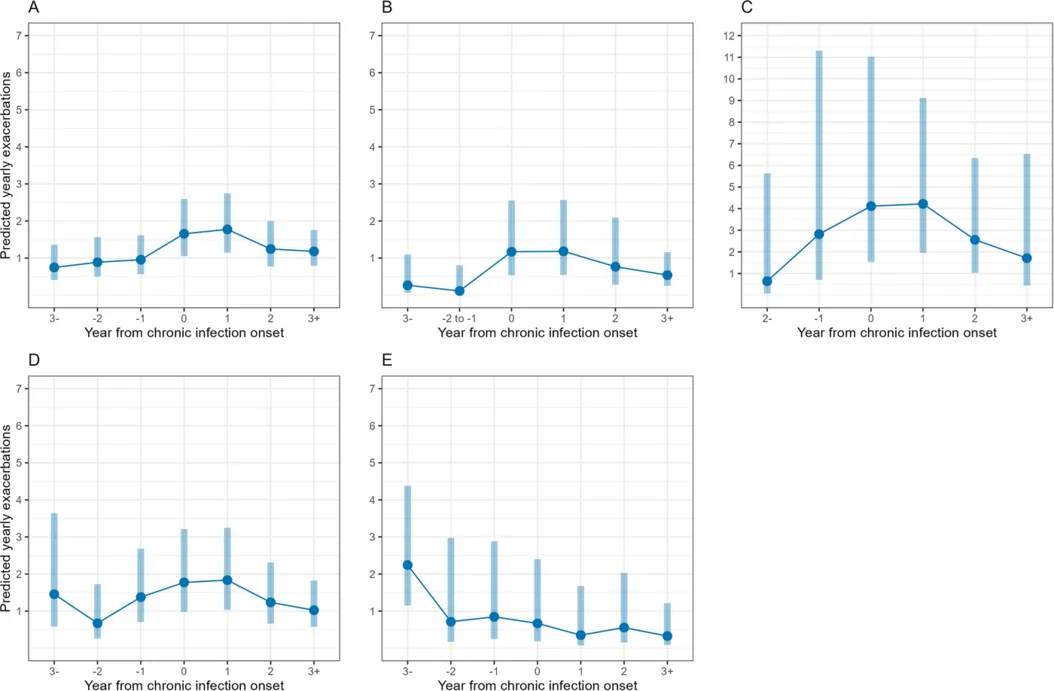
Predicted annual exacerbation rate within 3 years before and 3 years after onset of chronic infection. Data points (with 95% confidence interval) represent predicted annual exacerbation rate in patients using Poisson mixed effects models. A, any chronic infection; B, chronic *Pseudomonas aeruginosa*; C, chronic *Aspergillus fumigatus*; D, chronic *Haemophilus influenzae*; E, chronic methicillin‐sensitive *Staphylococcus aureus*. [Color figure can be viewed at wileyonlinelibrary.com]

The mean annual exacerbation rate was 1.7 (SD 1.2) among those with any chronic infection and 0.8 (SD 1.0) among those without chronic infections (*p* = 0.031, Kruskal‐Wallis test). The mean annual exacerbation rate was 0.7 (SD 0.4) among those with chronic PA infection and 1.5 (SD 1.3) among those without chronic PA (*p* = 0.184, Kruskal‐Wallis test). The ERR was significantly greater than 1 among subjects with any chronic infection, and those with chronic PA infection (Table 3). The risk for exacerbation was greatest approximately 1 year after onset of chronic infection but gradually returned to the baseline exacerbation rate approximately 3 years after onset of chronic infection (Figure 2).

Adjusted analyses accounting for IDA/MTD and chronic macrolide therapy yielded similar results. No significant difference in ppFEV_1_ was identified among subjects with chronic infections, with the exception of AF. The adjusted ERR for chronic infection was 1.71 (95% CI: 1.23–2.38); meaningful analysis of chronic PA infection was not possible given the small number of affected subjects. The five subjects with chronic PA infection harbored distinct genotypes (*DNAH5*, *DNAH11*, *CCDC40*, *CCDC65*, *CCDC114*). Biopsy‐confirmed IDA/MTD was present in one subject, two received chronic macrolide therapy, two had situs inversus totalis, and one had heterotaxy. Immune and splenic function were not assessed.

## 4 Discussion

In this single‐center, retrospective study of chronic airway infection in pwPCD, the majority (59%) had a chronic airway infection, including 16% with chronic PA. Chronic infection was not significantly associated with differences in ppFEV1, with the exception of chronic AF. Pulmonary exacerbations significantly increased after onset of chronic infections, particularly among those with chronic PA infection, with the most pronounced increase 1 year after onset of infection.

Prior studies of the impact of chronic airway infection in PCD have shown mixed results. In large multi‐center cohorts, individuals with PA in culture had lower FEV1 *Z*‐scores, particularly among adults [8, 10, 11, 12, 20]. Other studies showed no effect of PA on lung function [13, 21]. Little is known about the effect of chronic PA over time. A large longitudinal European study identified an overall mean reduction in FEV1 *Z*‐score of −0.06 when obtained in the proximity of positive PA cultures but the effect on the longitudinal rate of decline was not significant [22]. Whereas previous studies of chronic PA infection in cystic fibrosis (CF) identified a clear impact on longitudinal lung function, long‐term effects on lung function in PCD were not found in the current study and others [22]. Data on the long‐term impact of chronic PA are likely influenced by the variation in PA treatment regimens. In our study, among the 5 pwPCD with chronic PA infection, all received eradication treatment at the onset of infection, four were treated with chronic suppressive nebulized antimicrobial therapy, and two were treated with chronic macrolide therapy. Aggressive treatment of PA is a common practice at European PCD Centers and recommended by Better Experimental Approaches to Treat Primary Ciliary Dyskinesia (BEAT‐PCD), potentially explaining the trivial effect of PA on lung function seen at European centers [23].

Pulmonary exacerbations were defined in this study as respiratory tract symptoms leading to the initiation of systemic antibiotics excluding chronic antibiotics such as azithromycin or nebulized tobramycin. Annual exacerbation rates in this study were similar to exacerbation rates reported in other studies using similar definitions of pulmonary exacerbations [19]. The current study identified a greater rate of exacerbations in pwPCF after development of chronic PA infection. Similarly, a European study identified greater exacerbation rates and more severe bronchiectasis among patients with chronic PA infections but no significant difference in lung function [9].

Lung function was lower among the two pwPCD with chronic AF infection. Both pwPCD had a negative diagnostic workup for allergic bronchopulmonary aspergillosis (ABPA), a complex immune hypersensitivity reaction to AF reported among patients with obstructive lung diseases including CF. In CF, ABPA and non‐ABPA fungal infections have been associated with adverse clinical outcomes [24, 25]. However, besides rare case reports [26], very little is known about the effects of AF infections in PCD. Though limited by only two pwPCD, these results suggest a potentially important focus for future studies.

Chronic infection with HI was found in 10 pwPCD (31%) in our cohort and was not associated with significant differences in predicted ppFEV1. Other studies have similarly reported that HI does not lead to worsening lung function [21, 27]. Exacerbations rates were greater after chronic HI infection (ERR 1.29 [95% CI 0.72, 2.30]) but did not reach statistical significance (*p* = 0.388). Since HI is among the most common bacteria identified in sputum samples of patients with PCD [28] and 31% of pwPCD had chronic HI infection, the impact of chronic HI infection in pwPCD warrants further study.

Adjusted analyses accounting for factors expected to influence clinical severity, including IDA/MTD and chronic macrolide therapy, yielded results consistent with the primary analyses: chronic infection was not associated with impaired lung function but remained associated with a higher exacerbation rate. These analyses were limited by small sample sizes, and larger studies are warranted to better characterize the relationship between chronic infections and disease‐specific factors—including genotype, ciliary ultrastructure, and therapeutic interventions—in pwPCD.

Comparison of data across studies is limited by variable definitions of chronic infection/bacterial colonization. Research definitions of chronic infection/colonization in PCD differ considerably by the number of positive cultures, the length of time the infection exists and the interval of time between infections, compromising the ability to compare results [8, 9, 11, 20, 21]. Culture frequency is variable within and across PCD centers, particularly during the COVID‐19 pandemic, limiting the utility of encounter‐based criteria traditionally used in CF. However, annualized culture criteria (number of years with positive cultures) has demonstrated similar prevalence and duration of chronic PA as the more traditional encounter‐based culture methods in CF [29]. Therefore, to account for the variability in culture frequency and align with realistic clinical care, we adopted a modified version of a validated tool in which chronic infection is defined as at least 1 positive culture in 2 out of 3 consecutive years with at least 14 days between positive cultures [17].

The study is limited by a small sample size, particularly when analyzing individual cohorts of subjects with chronic infections. Lung function data were sparse 3 years prior to onset of infection. Exacerbation rates before and after infection, however, were more complete and suitable for predictive mixed effects modeling. In spring 2024, spirometry reference equations were changed from the Third National Health and Nutrition Examination Survey (NHANES III) equations to Global Lung Function Initiative (GLI) standards. Given that data collection ended shortly thereafter, this transition is unlikely to have substantially influenced ppFEV_1_ outcomes. The incidence of pulmonary exacerbations in CF decreased during the COVID‐19 pandemic for multiple reasons, including reduced clinical exposures, masking, and social distancing [30]. Our estimates of annual exacerbation rates during this time period, therefore, might have inadequately captured a typical exacerbation rate. However, the study was specifically designed to evaluate differences in exacerbation between patients with and without chronic infection, not the absolute rate per year. The study was also limited by the variability in care between pediatric and adult patients. However, whereas 12/32 subjects in the cohort were adults over the age of 18 at the time of the study, 29/32 were managed in the pediatric clinical center, ensuring relatively consistent clinical practices. Lastly, the impact of infections is incomplete without an analysis of co‐infections and immune function, particularly in patients with heterotaxy. Larger multi‐center studies would be required for this analysis.

In conclusion, this study explored clinical outcomes in people with confirmed PCD and chronic airway infection in the context of contemporary antimicrobial prescribing practices at a PCD Foundation Clinical and Research Center using a practical definition of chronic infection. Chronic infection with AF was associated with a significant difference in lung function. Chronic infection with PA was associated with a greater rate of pulmonary exacerbations. Larger, multi‐center studies, with the future PCD Foundation Registry, are needed to thoroughly explore the long‐term clinical outcomes. The impact of chronic infections found in this study suggests a need to re‐evaluate the infection prevention and control guidelines at PCD Foundation Clinical and Research Centers to reduce transmission of infections with potentially adverse clinical outcomes. This study, and others, provide a framework for future prospective studies and, when considered alongside expert consensus and standardized guideline development, may ultimately contribute to improvements in clinical care.

## Author Contributions

**Thomas G. Saba:** conceptualization, investigation, funding acquisition, writing – original draft, methodology, validation, visualization, writing – review and editing, formal analysis, project administration, data curation, supervision, resources. **Madsen Zimbric:** conceptualization, investigation, methodology, validation, writing – review and editing, formal analysis, data curation, resources. **Harlan McCaffery:** methodology, writing – review and editing, formal analysis, data curation. **Lindsay J. Caverly:** conceptualization, investigation, funding acquisition, writing – original draft, methodology, validation, visualization, writing – review and editing, formal analysis, project administration, data curation, supervision, resources.

## Ethics Statement

The study was deemed exempt by the University of Michigan Institutional Review Board.

## Conflicts of Interest

The authors declare no conflicts of interest.

## Scientific Meetings

Study results were presented in the form of a poster at the PCD On the Move conference in Minneapolis, Minnesota in August 2025.

## Data Availability

The data that support the findings of this study are available on request from the corresponding author. The data are not publicly available due to privacy or ethical restrictions.
